# Effects of Risk and Time Preferences on Diet Quality: Empirical Evidence from Rural Madagascar

**DOI:** 10.3390/foods13193147

**Published:** 2024-10-02

**Authors:** Sakiko Shiratori, Mudduwa Gamaethige Dilini Abeysekara, Ryosuke Ozaki, Jules Rafalimanantsoa, Britney Havannah Rasolonirina Andrianjanaka

**Affiliations:** 1 Japan International Research Center for Agricultural Sciences, 1-1 Ohwashi, Tsukuba 305-8686, Japan; 2 The National Office of Nutrition of Madagascar, Madagascar, Lot III M 39, Avenue Dr Joseph Ravoahangy Andrianavalona Anosy, Antananarivo 101, Madagascar

**Keywords:** dietary diversity, Madagascar, risk preference, time preference

## Abstract

Malnutrition is a major concern in Madagascar. Eating a wide variety of nutritious food is necessary because Malagasy diets heavily rely on rice consumption. This study explored the barriers to dietary change towards diversification from the perspective of consumer behaviour. We analysed the impact of risk and time preferences on dietary diversity using economic experiments conducted with 539 rural lowland rice farmers in Central Highlands in Madagascar. The results showed that risk-averse or impatient individuals were more likely to have lower Household Dietary Diversity Score (HDDS), indicating poorer diet quality. Risk-averse people may not want to add different foods to meals as they perceive unfamiliar food as a ‘risk’; people who prefer immediate gratification may fail to invest in nutritious diets now to achieve better health in the future. Additionally, higher HDDS was observed among households with a female head who earned off-farm income and who had frequent market visits. These findings contribute to explaining the limited shift in nutritional transition in Madagascar and provide useful insights into nutritional policies promoting healthier food choices. Depending on the preferences, more focused support such as nutritional education, financial support, market development, and pre-commitment mechanisms could be provided to reward long-term nutritional benefits.

## 1. Introduction

Nutritional improvement has been prioritised in many domestic and international policy agendas, including the Sustainable Development Goals. Since a key risk factor for all forms of malnutrition and diet-related diseases is poor dietary habits [1,2], understanding the underlying motivations for food-choice decisions is critical for the development of effective health policies. 

Diet choice is largely related to various competing, interacting, and reinforcing economic, social, and political factors. In the empirical literature, consumer demand for different foods is often explained by price, product characteristics, and income [3] while treating preferences as heterogeneous to an individual. However, there is substantial evidence supporting the notion that behavioural economics is a promising approach to encouraging healthy food choices [4]. 

Increasing evidence shows that risk and time preferences, which are elements of behavioural economics, play an important role in diet choice [5,6]. An individual’s diet choice is accompanied by delayed or uncertain health consequences [7]. We may assume that risk-averse individuals may not want to adopt new diets or change their dietary behaviour. In contrast, risk-seeking individuals may be willing to divert from their usual routines. Regarding time preference, people who tend to discount the future may fail to invest in nutritious diets ‘now’ to achieve good health ‘in the future’. 

In this way, we hypothesise that experimental choices of risk and time preferences are related to healthy eating behaviour measured through the likelihood of adopting a diverse diet. Dietary diversity is a measure of diet quality because it reflects nutrient adequacy [8]. For example, when people diversify their staple diets with more fruits, vegetables, animal-sourced foods, and dairy products, they increase their essential micronutrient intake [9].

Previous research shows the benefits of examining the nexus of behavioural economics and health behaviours. For instance, the relationships between risk and time preferences and healthy or risky behaviour [10,11,12,13,14,15,16] and health outcomes [17,18,19,20,21,22,23,24] have been extensively studied. However, similar evidence on the factors shaping diet choice is relatively limited, as summarised in Supplementary Table S1. Many of these studies are confined to the developed world, yet economic preferences exhibit substantial heterogeneity [25]. While basic nutrition is prioritised in poor economies, promoting healthy nutrition and behaviours to mitigate health risks (i.e., obesity) and sustain economic well-being is the focus in wealthy economies [26]. These observations require new interpretations in the context of developing countries. No prior research has explored the economic determinants underlying dietary choices in the context of this study, and empirical evidence is scarce.

Madagascar, a sub-Saharan African country, has an ‘alarming’ number of nutritional deficiencies. The prevalence of undernourishment in the population increased from 33.7% in 2004–2006 to 51% in 2020–2022, with the latter value being nearly double the average value for low-income countries [27]. Madagascar is in a very early stage of nutritional transition [28,29]. Almost monotonous energy-rich staple-based diets continue to characterise Malagasy cuisine, which features a large portion of rice accompanied by smaller portions of side dishes (laoka). The side dishes are primarily composed of vegetables and occasionally include fish or meat. Households often prioritise the quantity of rice consumed and pay little attention to a diverse diet. Notably, the country faces a substantial need for improved nutrition through healthy dietary choices. 

Global evidence suggests that one of the main reasons for malnutrition is food scarcity, which may arise due to geographical or economic inaccessibility [27]. However, in Madagascar, food scarcity cannot be considered the prime reason for malnutrition because a large variety of natural resources, which could potentially be sufficient to feed the entire population, are available [30]. Although nutrient-dense low-cost foods, such as fruits, leafy vegetables, and legumes, are available and affordable in Madagascar, they are not often readily consumed [31,32,33,34], especially by the poorest households [31].

Another factor responsible for the Malagasy diet could be poverty, but poverty alone cannot explain their dietary choices. Rice is the most consumed food by Malagasy people regardless of their economic status, although it is not the most affordable carbohydrate staple [31]. Previous studies have attempted to investigate the diet choices of Malagasy people arising from nutritional and cultural beliefs and food practices [31,32,35], availability of alternative food sources [36,37], and the effect of different awareness approaches [34,38]; however, the understanding of why low-cost nutrient-dense locally available food is not readily consumed by Malagasy people, and what may be the underlying reason for a diet choice that is biased towards rice, remains limited.

This study aims to fill this gap by exploring the relationship between diet choices, as reflected in diverse diets, and risk and time preferences, using the originally collected data from rural Madagascar. We explore the reasons behind food choices and identify the factors that make changing dietary habits in Madagascar difficult from the perspective of consumer behaviour. This study contributes to the growing literature by suggesting the possible association between diet choice and economic preferences and also contributes to policy to provide more focused support for nutritional improvement by identifying behavioural characteristics. 

## 2. Materials and Methods

### 2.1. Data Collection

This study used a subset of the household data collected for the FertilitY sensing and Variety Amelioration for Rice Yield (Fy Vary) project conducted jointly by the Japan International Research Center for Agricultural Sciences and the Ministry of Agriculture, Livestock, and Fisheries in Madagascar. We used data collected in January and February 2022, which correspond to the period before the main harvest season. The project site was the Vakinakaratra region in the Central Highlands in Madagascar, where 93% of the households are engaged in agriculture and 87% of them are rice farmers [39]. Three of the seven districts in the region were selected for sampling considering the access to the main road. In proportion to the size of each district, we selected 60 villages that ensure wide geographical variation, from which we then randomly selected 10 lowland rice-farming households. The selection yielded an initial sample of 600 households. 

The main survey collected detailed information on various topics, including household demographics; socioeconomic information, including agricultural activities, off-farm income generation, household and farm assets, and monthly expenditure on food and non-food items; dietary habits at the household level; and 24 h dietary recall. Economic experiments were incorporated into the main survey. Of the 600 households who participated in the main survey, 539, particularly the head of the household or the person responsible for decision-making in the household, participated in the experiments. Informed consent was obtained from all participants. Ethical approval for this study was obtained from the Malagasy Ethical Committee for Science and Technology (N°014/2020-AM/CMEST/P).

### 2.2. Economic Experiments

Risk and time preferences were measured using hypothetical economic experiments designed to be sufficiently simple for participants to understand and allow us to estimate the desired preference parameters with theoretical consistency. The hypothetical approach is often used in field surveys to minimise costs and other associated logistic limitations, resulting in the same risk preference profiles as real choices and making no difference in the field [40,41]. A more straightforward task may be preferred when participants exhibit low numeracy as it generates less noisy behaviour but has similar predictive accuracy to a more complex task [42,43]. As our focus was on the relationship between dietary diversity and economic preferences rather than on estimating the average levels of risk aversion and patience in the study population, we measured preferences in terms of row-switching points in both experiments to eliminate any confounding effects that may arise due to parametric assumptions. The values of the choices were determined using several pretests. The choices provided in the experiments are listed in Table 1 and Table 2.

As shown in Table 1, the participants were given a choice between a guaranteed payoff and a 50/50 lottery. In the lottery, the participants were asked to imagine that they were going to pick one ball from a bag containing two differently coloured balls. If the colour of the ball was pink, they could win 8000 MGA. However, if it was green, they would lose and receive only 800 MGA; each option had a 50% probability. If the participants were unwilling to play, they would receive the corresponding payoff, the amount of which increases along the row in Table 1. Following Dohmen et al. [44], the participants were coded as either risk-averse or risk-seeking based on the corresponding row-switching point. As the expected value of the lottery was 4400 MGA, a risk-averse participant would prefer guaranteed payoffs equal to or smaller than 4400 MGA over the lottery (i.e., switching before the seventh row). Similarly, only a risk-seeking participant would prefer the lottery over guaranteed payoffs greater than 4400 MGA (i.e., switching at or after the seventh row). The risk preference measure used in the analysis was a binary variable indicating whether the participant was risk-averse or risk-seeking. 

For time preference, the participants were asked to choose between money ‘today’ and a more considerable amount of money ‘one month later’. As Table 2 shows, today’s payoff decreases for each consecutive question. The corresponding row number in which the participants switched from today to later indicated their time preference. (Some participants are more impatient even in the final row of the choice table). We categorised the participants into three groups based on the distribution of the row-switching points. Participants who selected to switch during the earlier stage (≤4th row) were considered patient participants; those who shifted between 5th and 7th rows were categorised as moderately patient; and those who shifted in >8th row and never switched were categorised as impatient. 

To account for other individual- and household-level determinants that might be associated with dietary diversity, we included control variables such as individual characteristics (gender, age, and level of formal education of the respondent), household characteristics (i.e., gender of the household head, household size, dependency ratio, land size, involvement in off-farm income generation activities, frequency of market visits, and monthly consumption expenditure), and farm characteristics (e.g., crop diversification and livestock ownership). The variable definitions and their means/proportions are provided in Section 3.

### 2.3. Empirical Estimation

A variety of methodologies have been used to see the relationship between economic preferences and health/healthy behaviour. For time preferences, proxies included questions on monetary or non-monetary intertemporal choices, which can be either hypothetical or incentivised [42,45], general self-reported measures based on agreement with statements related to patience [46,47], information on savings, financial planning horizons, and so on. Choice tasks, in which the participants choose between a smaller but more immediate award and a more desirable delayed award, represent the most common and typically used approach. These choices are either made in the monetary or non-monetary domain and then compared to actual health-related behaviour [14]. Choosing which method to utilise in eliciting economic preferences, however, is largely dependent on the research question and the characteristics of the sample population [48]. 

Excessive and unhealthy eating is most commonly assessed using body mass index (BMI) as an indicator [17,18,19,20,22,23,24,47]. Very few studies have estimated the overall diet quality in relation to risk or time preference [7,46,49]. The measurement of diet quality is usually difficult in low-income settings, where a lack of resources and a high level of technical capacity for collecting and analysing detailed dietary data co-exist [50]. In such contexts, dietary diversity (DD), the number of food groups consumed which has long been recognised as a key element of a high-quality diet, can be used in operationalising the diet quality. To either assess the economic access to food or to estimate the food groups a particular household is consuming, the Household Dietary Diversity Score (HDDS), a population-level indicator used as a proxy measure of household food access, is an appropriate tool [8,51]. 

The 12 food groups used to calculate the HDDS were cereals; roots and tubers; vegetables; fruits; meat, poultry, and offal; eggs; fish and seafood; pulses, legumes, nuts; milk and milk products; oil/fats; sugar/honey; and miscellaneous [8,51]. Each food group was assigned a score of 1 if it was consumed over the previous 24 h or 0 otherwise. The HDDS ranges from 0 to 12 and is equal to the total number of food groups consumed by the household. To consider the dietary patterns of the households at different HDDS levels, the households were categorised into three groups based on HDDS tertiles, which are commonly used for analytical purposes [8,51,52]. Based on the HDDS distribution, the three categories were low (HDDS ≤ 5), middle (HDDS = 6), and high (HDDS ≥ 7). 

The basic equation that we estimate is given by Equation (1).

*Y_h_* = *β*_1_ Risk preference *_h_* + *β*_2_ Time preference *_h_* + *β*_3_
*X_h_* + *e* (1)

$Y_{h}$ represents the dependent variable, which can be the HDDS of the *h*th household. Risk preference and Time preference, the main explanatory variables of interest, correspond to the risk and time preference measures estimated through the experiments. $X_{h}$ is the vector of variables of the *h*th household, and e captures the error. 

We conducted ordinary least squares (OLS) analyses for the HDDS. In addition, ordered logistic regression was specified for the categorised HDDS. For the OLS model, the kernel density plot, interquartile range test for normality, and Shapiro–Wilk W test for normal data indicated that the residuals had an approximately normal distribution. According to the multicollinearity test, the mean-variance inflation factor (VIF) value was 1.86, while the individual VIF values were lower than 5, indicating no collinearity among the predictors. For the ordered logistic regression model, we conducted the Brant test. We found that while one variable (frequency of market visits, *p* < 0.1) violated the underlying proportional odds assumption in ordered logistics, the Akaike information criterion and Bayesian information criterion made strong cases favouring the estimated ordered logit model over the other two alternative models, namely, the multinomial logit and generalised logit models.

## 3. Results

### 3.1. Descriptive Statistics

Table 3 presents the summary statistics of the key-dependent and independent variables. On average, the HDDS for our sample was 5.68, which is similar to that estimated in the previous literature on Malagasy dietary diversity, as presented in Supplementary Table S2. The participants were primarily middle-aged (47.04 ± 14.22 years) men (51%) having at least some formal education (5.14 ± 3.61 years of schooling). Among the participants, 42% were risk-averse. Most of the households were male-headed (88%). The average household size was 4.5. We used total household consumption expenditure as a proxy variable in the absence of accurate monthly household income data. The average monthly household income expenditure was approximately 250,000 MGA, or approximately 63 USD, during the survey period. Most households (89%) had at least one member who had engaged in off-farm income generation activities during the last two months. We also included a binary control variable to identify whether the respondent was primarily responsible for preparing meals to reduce possible measurement errors in the consumption questionnaire. Almost half of the respondents were not primarily responsible for household meal preparation and consumption.

We generated a crop diversification indicator that represented the number of crop species (excluding rice) produced on the farm. On average, the households produced at least one crop other than rice. We determined the livestock ownership of a household by calculating the total livestock unit (TLU), which allowed us to compare different livestock herds. The estimated average TLU per household was 2.03. Most respondents visited local markets at least weekly. In the context of this study, the term ‘local market’ refers to a nearby village or communal market that primarily sells food and daily essentials, such as oil, sugar, and salt.

### 3.2. Estimation

**Figure 1:**
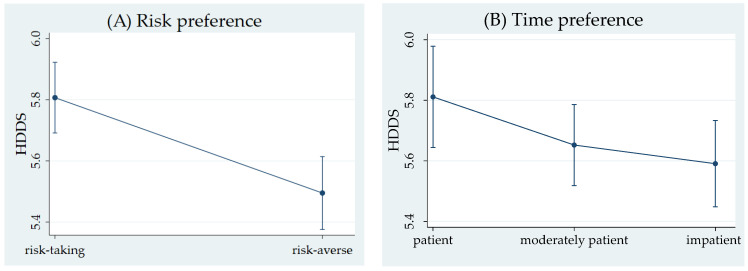
Predictive margins with 95% CIs for risk (**A**) and time preference (**B**) on HDDS.

Table 4 presents the results of the econometric estimations. The ordered logistic regression results of the HDDS categories are reported as adjusted odds ratios (ORs) for moving from one category to the next versus remaining in the same category. Regardless of the model specification, risk aversion was associated with a low HDDS. Figure 1 illustrates the predictive margins with 95% confidence intervals for risk and time preference on HDDS based on Model 1. The results of Model 1 suggest that impatient individuals are more likely to have lower HDDS. Therefore, respondents who were more risk-averse and less patient were likely to be in the lower category of the HDDS. Moreover, the frequency of market visits, involvement in off-farm income generation activities, and the total value of assets owned by households were positively associated with HDDS. These findings are robust across alternative model specifications. 

As the ordered logistic regression model identifies the natural ordering of dependent variables, it further allows the estimation of the marginal effect for each independent variable and category of dependent variable pairing. According to Table 5, the marginal effects of Model 2 indicate that the likelihood of being in the low HDDS category increases by 16 percentage points and the likelihood of being in the middle and higher HDDS categories decreases by 5 and 11 percentage points, respectively, if the individual becomes risk-averse instead of risk-seeking, all else remaining constant. Furthermore, on average, impatient individuals are 13 percentage points more likely than patient individuals to be in the low HDDS category and approximately 9 percentage points less likely to be in the high HDDS category. 

## 4. Discussion and Policy Implications

### 4.1. Discussion

Our findings indicate that risk-averse and impatient individuals are likely to have lower HDDS. Previous studies have found that risk aversion and patience are negatively associated with risky health behaviours [14,21,22,54]. Our finding on the time preference measure is consistent with the previous literature, confirming our hypothesis that people who prefer immediate gratification may fail to invest in nutritious diets now to achieve good health in the future. 

If we consider monotonous diets as a risky health behaviour that could cause malnutrition, our findings on risk aversion may seem to be contradictory to previous literature. Nevertheless, our findings may still be intuitive, given the context. Malagasy people are accustomed to eating traditional rice-biased food and may thus perceive unfamiliar food as a ‘risk’. In this case, risk-seeking behaviour indicates their willingness to include unfamiliar food in their diet. 

These risk and time preferences, elements of behavioural economics, are behind their diet choices in the context of developing countries. From the perspective of consumer behaviour, the preference of risk-averse and impatience could be one of the factors that makes changing dietary habits in Madagascar difficult. A previous study showed that the sub-Saharan population is among the most risk-tolerant countries, and on average the population deviates from the world mean on patience and they are rather impatient [25]. 

Frequent market visits were found to be associated with increased household diversity, and this finding is in line with other studies [55,56]. The market can provide farmers with not only increased access to diverse food items but also income opportunities [57]. The involvement of household members in off-farm income generation activities also contributed to increased dietary diversity. Household cash income generated from farm production and off-farm labour is an essential driver of food access in smallholder farm households [55]. As the survey was conducted just before the harvesting of the main crop (i.e., rice), the farmers might have had limited consumable and marketable farm produce. Farmers’ risk preference not only affects their diet choices but also has an impact on their inter-temporal food sales. A higher risk perception leads to a greater likelihood of favouring current sales over intertemporal ones [58]. This can indirectly affect their household cash income during lean periods, leading to limited food choices for the household during such times. 

The gender of household decision-makers also has a significant association with diverse diets. In our study, male respondents were less likely to contribute to increased dietary diversity in the household. In traditional rural societies, women typically cook and make household food choices. Notably, women tend to prioritise a wider variety of food options available in households [59]. Although this study provides valuable insights, it has several limitations. First, our outcome measure is the HDDS, which is a household-level measurement. Having data on individual-level food consumption would enable us to deepen our findings further because preference measurements are inherent in individual behaviour. Second, the economic experiments used in this study have an established history; however, considering the participants’ very low income and financial literacy, their choices in the economic experiments might have been influenced to a greater extent by immediate financial concerns (as we observed, especially in the time preference experiment) than we might expect in a more well-off population. Third, in consideration of the contextual dependency of experimental findings, future research endeavours must assess the resilience of our present conclusions across diverse contexts, encompassing varying preference measures and geographic locations.

### 4.2. Policy Implications

We found that risk aversion and impatience observed in this study may be the barriers to household dietary diversification. To the best of our knowledge, our work is one of the few studies to examine whether economic preferences are related to diet choices, particularly in developing countries. The population studied was of particular interest because of minimal or no nutritional transition observed [28] and a higher rate of chronic nutritional deficiencies [27] amid various policy interventions. 

Our findings emphasise the importance of integrating behavioural economics tools in analysing household food choice behaviour and the policy for promoting healthier food choices. Unlike many sociodemographic characteristics, economic preferences are flexible; that is, they can be changed through behavioural choices [60,61,62]. Although consumer behaviour perspectives emphasise that dietary choices are rarely considered in nutritional policy interventions, empirical evidence suggests that the use of incentives is effective in promoting changes in dietary behaviour [63]. Combining healthy food subsidies with behavioural interventions (i.e., changes to the choice environment) is effective in modifying health behaviours such as healthy food spending and dietary habits [64].

By identifying the risk-averse and impatient individuals, more focused support for nutritional improvement can be provided. For risk-averse people, a policy to create an environment where people can perceive that adding unfamiliar foods to their diets is safe and rewarding would be necessary. Nutritional education emphasising the health benefits of a diverse diet and recipe introduction through cooking demonstrations, school lunches, or media to get familiar with the new food could contribute to it. Moreover, enhancing access to diverse foods through financial support, such as subsidies for healthy food, or the development of well-functioning local markets that offer diverse foods at affordable prices, would be beneficial, especially for people in poverty. Individuals with limited resources may face higher risks when attempting behavioural changes because they have fewer means to mitigate potential adverse consequences. In this way, price instruments, one of the traditional policy tools, could be effective.

For impatient people, a policy to inform the connection between their current daily consumption and their health in the long run would be needed. Enabling individuals to consider the future could serve as an effective strategy for mitigating unhealthy food consumption among those who are particularly susceptible to this behaviour (i.e., individuals who do not try restricting their caloric intake, and individuals with a higher BMI) [65]. In addition, pre-commitment mechanisms could be applied to adhere to their long-term health benefits. For example, nutritional education can provide pre-planned menus or can include a curriculum for them to plan menus for one week. Pre-planned menus and pre-planned expenditures (budget for nutritious foods) are kinds of commitments. Furthermore, if they have social groups, they can share information and support mutually, which could reinforce commitment. Such mechanisms facilitate more thoughtful consideration of choices for less patient individuals so that they can better weigh decisions that yield long-term health benefits. 

In addition to preference measures, our finding thus highlights the importance of empowering women in household decision-making, which can lead to dietary diversification. Markets and non-farm income generation also contribute to household dietary diversity, especially when few or no food crops are produced locally. Therefore, strengthening the inter-regional agro-marketing network, which can facilitate the distribution of surplus produce, is imperative to ensure food availability.

## 5. Conclusions

Nutritional deficiencies, which arise partly from less diverse diets, are a significant health concern in Madagascar. Despite global trends, the country is not experiencing a noticeable nutritional transition, and people do not seem willing to divert from their traditional diet, which is biased towards energy-rich staple foods. Using a dataset collected from 539 households in central Madagascar in 2022, this study explored the behavioural factors associated with household dietary diversity, focusing on risk and time preferences. In rural Madagascar, risk aversion and impatience may be the barriers to household dietary diversification. Our insights from behavioural economics contribute to the development of effective intervention strategies to promote dietary diversity and divert the direction of nutritional policies towards nutritional improvement. 

## Figures and Tables

**Table 1:** Experiment for risk preference.

Decision	Option A (Sure Payoff) MGA *	Option B (Lottery) MGA	Expected Payoff Difference (MGA)
1	500	½ of 8000, ½ of 800	−3900
2	1000	½ of 8000, ½ of 800	−3400
3	1500	½ of 8000, ½ of 800	−2900
4	2000	½ of 8000, ½ of 800	−2400
5	3000	½ of 8000, ½ of 800	−1400
6	4000	½ of 8000, ½ of 800	−400
7	5000	½ of 8000, ½ of 800	600
8	6000	½ of 8000, ½ of 800	1600
9	7000	½ of 8000, ½ of 800	2600
10	8000	½ of 8000, ½ of 800	3600

* Malagasy Ariary (MGA) is the Malagasy currency. At the time of the survey, 1000 MGA = 0.2516 USD (monthly average of February 2022; FAOSTAT).

**Table 2:** Experiment for time preference.

Decision	Choice A_Payoff Today (MGA)	Choice B_Payoff One Month Later (MGA)	Implicit One-Month Discount Factor (If Indifferent)	% Choosing Choice B
1	50,000	50,000	1.0000	1.26
2	40,000	50,000	0.8000	7.58
3	30,000	50,000	0.6000	9.75
4	20,000	50,000	0.4000	13.72
5	15,000	50,000	0.3000	12.64
6	10,000	50,000	0.2000	8.48
7	8000	50,000	0.1600	9.93
8	5000	50,000	0.1000	8.30
9	1000	50,000	0.0200	20.94
10	200	50,000	0.0040	0.36
Never switch				7.04

**Table 3:** Descriptive statistics of the variables (n = 539).

Variable Description	Mean/Prop. (SD)
Panel A: Outcome variables	
HDDS	5.68 (1.03)
HDDS category	
Low (≤5 food groups)	0.46
Middle (=6 food groups)	0.34
High (≥7 food groups)	0.20
Panel B: Explanatory variables	
Risk preference (=1 if the participant is risk-averse and 0 otherwise)	0.42
Time preference	
Patient (≤4)	0.28
Moderately patient (5–7)	0.40
Impatient (≥8)	0.32
Gender of the respondent (=1 if male and 0 otherwise)	0.51
Age of the respondent (years)	47.04 (14.22)
Level of formal education obtained by the respondent (years of schooling)	5.14 (3.61)
Gender of the household head (=1 if male-headed and 0 otherwise)	0.88
Number of household members	4.48
Household monthly consumption expenditure (‘000 MGA)	250.75 (107.14)
Off-farm income-generating activities in the last two months (=1 if at least one member is involved)	0.89
Total land size (ha)	0.47 (0.79)
Total lowland rice area (ha)	0.37 (0.78)
Livestock owned by household (tropical livestock unit: TLU) ^a^	2.03 (2.13)
Respondent for the consumption questionnaire (=1 if the respondent is different from the person who knows the most about household food consumption)	0.48
Ratio of children under the age of 5 in the household	0.08 (0.13)
Ratio of members aged 65 years or above in the household	0.08 (0.22)
Value of household and farm assets ^b^ owned by the household (‘000 MGA)	530.44 (972.07)
Frequency of market visits	
Everyday	0.12
At least weekly	0.57
At least monthly	0.31

^a^ TLU describes livestock numbers across species to produce a single figure indicating the total ‘amount’ of livestock owned. Conversion equivalents of sub-Saharan African livestock into TLU were adapted from Njuki et al. [53], with a mature cow (calved > once) weighing 250 kg equivalent to 1 TLU. ^b^ Household assets include 19 non-productive assets, such as radios, refrigerators, TVs, bicycles, cell phones, chairs, tables, and beds. Farm assets include 21 productive assets, such as plough sets, carts, wheelbarrows, tractors, and weeders, excluding livestock.

**Table 4:** Regression results of determinants of HDDS.

Variables	(1) OLS: HDDS		(2) Ordered Logit: HDDS Category	
Gender of the respondent	−0.34 **	(0.17)	−0.69 *	(0.38)
Age of the respondent in years	−0.00	(0.00)	−0.00	(0.01)
Years of schooling completed by the respondent	−0.00	(0.01)	−0.01	(0.03)
Risk preference (risk-averse)	−0.31 ***	(0.09)	−0.76 ***	(0.18)
Time preference (patience = omitted)				
Moderately patient	−0.16	(0.11)	−0.40 *	(0.22)
Impatient	−0.22 *	(0.12)	−0.60 ***	(0.24)
Male-headed household	0.16	(0.15)	0.42	(0.31)
Number of members in the household	−0.02	(0.03)	−0.07	(0.06)
Market visits (everyday = omitted)				
At least once a week	−0.19 *	(0.11)	−0.39 *	(0.21)
At least once a month	−0.36 ***	(0.10)	−0.89 ***	(0.22)
Logarithm of monthly household consumption expenditure	0.16	(0.15)	0.15	(0.27)
Involvement in off-farm income generation in the last two months	0.29 **	(0.13)	0.84 ***	(0.32)
Household-owned total land size (ha)	0.04	(0.06)	0.10	(0.11)
Tropical livestock unit (TLU) owned by household	0.02	(0.02)	0.06	(0.04)
Respondent is different from the person who knows most about household consumption	0.19	(0.16)	0.42	(0.36)
Ratio of children under the age of 5 in the household	0.09	(0.36)	0.44	(0.70)
Ratio of members aged 65 years or above in the household	−0.03	(0.27)	0.05	(0.55)
Logarithm of the total value of household assets	0.11 **	(0.05)	0.18 **	(0.09)
/cut1			3.43	(3.06)
/cut2			5.18 *	(3.06)
Constant	2.62	(1.64)		
Observations	539		539	
R-squared/Pseudo R-squared	0.14		0.08	

Note: Robust standard errors in parentheses; *** *p* < 0.01, ** *p* < 0.05, * *p* < 0.1.

**Table 5:** Marginal effects of risk and time preferences on HDDS category.

	Low HDDS	Middle HDDS	High HDDS
Risk preference			
Risk-averse	0.16 *** (0.04)	−0.05 *** (0.01)	−0.11 *** (0.03)
Time preference (Patient-based outcome)			
Moderately patient	0.08 * (0.05)	−0.02 * (0.01)	−0.06 * (0.04)
Impatient	0.13 *** (0.05)	−0.04 ** (0.01)	−0.09 ** (0.04)

Note: Robust standard errors in parentheses; *** *p* < 0.01, ** *p* < 0.05, * *p* < 0.1.

## Data Availability

The original contributions presented in the study are included in the article/Supplementary Material, further inquiries can be directed to the corresponding author.

## Supplementary Materials

The following supporting information can be downloaded at https://www.mdpi.com/article/10.3390/foods13193147/s1, Table S1: Summary of selected studies on the effect of economic preferences on health, health behaviour, and diet quality; Table S2: Summary of selected studies on dietary diversity estimates in Madagascar; and Supplementary data. Supplementary tables refer to [7,14,15,17,18,19,21,22,23,24,34,37,42,45,46,47,49,66,67,68,69,70,71,72,73,74,75,76] in the references.
