# Supplementary material for: Effects of Risk and Time Preferences on Diet Quality: Empirical Evidence from Rural Madagascar
Source: Foods. 2024 Oct 2;13(19):3147. doi: 10.3390/foods13193147 (PMC11476199; doi:10.3390/foods13193147)
Supplement: Supplementary file 1 [file foods-13-03147-s001.zip › 20241001 foods-3191272 supplementary tables_edited submitted.pdf]

Supplementary Table S1. Summary of selected studies on the effect of economic preferences on health, health behaviour, and diet quality

Approach	Elicitation method	Study	Sample	Summary
**Panel A:** Time preference and health outcome and food choice	Questionnaire	[46]	Adults aged 20 years and older/United States	Proxies for future discount rates influence diet choices measured by the Healthy Eating Index (HEI).
	Questionnaire	[17]	Young adults/United States	Proxies for time preferences are positively related to body mass index (BMI) in nationwide youth survey.
	Questionnaire and selection of immediate/delayed reward	[18]	Dutch population aged 16 years and older	Proxies for discount rates significantly predict BMI in cross-sectional analysis.
	Selection of immediate/delayed reward	[19]	High school graduates/United States	Individual discount rates better predict inter-individual variation in field behaviours (e.g. exercise, BMI, smoking) than demographic variables (e.g. sex, age, education).
	Consideration of Future Consequences scale (CFC)	[66]	Dutch adults aged 18–60 years	Behaviour-specific individual differences in time orientation contributes to self-reported healthy eating but not the proxies for discount rate.
	Selectin of immediate/delayed reward	[15]	Adults aged 20–65 years/Japan	Health-related measures (smoking, health condition, etc.) show strong associations with the degrees of impatience and declining impatience.
	Selection of immediate/delayed reward	[45]	Adults/United States	Higher future time preferences relate to a lower frequency of fast-food consumption.
	Selection of immediate/delayed reward and CFC	[67]	Adolescents aged 16–19 years and parents/United States	Higher patience is significantly associated with lower BMI and self-reported lower consumption of fast foods and sweets.
	Immediate/delayed reward	[68]	Low-income shoppers/United States	There exists a direct link between key food choice behaviours and incentivised measures of patience and hyperbolicity.
**Panel B:** Risk preference and health outcome and food choice	Selection of lottery	[69]	Undergraduates/United States	Risk preference and risk perception measured in a lab setting are significant determinants of acceptance of genetically modified food elicited by a survey.
	Selection of lottery	[14]	Students and non-student adults/United States	Risk aversion is negatively and significantly associated with cigarette smoking, heavy drinking, and being overweight or obese.

	Selection of lottery	[49]	Young adults/United Kingdom	For males, risk preference is positively associated with BMI and the Healthy Eating Index.
	Staircase risk	[70]	Indian households	Risk preference positively influences dietary diversity.
**Panel C:** Economic preferences and health outcome/ food choice	Time: Selection of immediate/delayed reward<br>Risk: Selection of lottery	[21]	Adults in low-income, urban, African American neighbourhoods	Individuals who are more tolerant of financial risks and more patient are more likely to be in a more advanced physical activity stage.
	Time: Selection of immediate/delayed reward<br>Risk: Two-colour choice task	[22]	Children and adolescents aged 10–18 years	More impatient adolescents are more likely to drink and smoke and have high BMI and experimental measures for risk weekly predict field behaviour.
	Time: Acceptance/rejection of delays<br>Risk: Acceptance/rejection of lottery	[42]	Children and adults in poor households/ Cambodia	Risk-taking households have taller and heavier children. Impatience does not affect child health but is positively and moderately correlated with adults' BMI.
	Time: Selection of immediate/delayed reward<br>Risk: Selection of lottery	[23]	Adults in low-income, urban, African American neighbourhoods	Individuals who are more tolerant of risk are more likely to have a higher BMI and be more risk-averse, while patient individuals are less likely to be obese.
	Risk and Time: Questionnaire	[47]	Metropolitan households/ Thailand	Risk-averse individuals are more likely to be obese and tend to have a higher BMI. Proxies for time preference do not predict BMI in the Thai context.
	Time: Selection of immediate/delayed reward<br>Risk: Selection between lottery and guaranteed payment	[24]	Adults with an average age of 55 years/Australia	A significant negative association exists between risk tolerance and BMI and other markers of obesity for women. Impatience moderates the link between risk tolerance and obesity.
	Questionnaire	[7]	A representative sample of the French population	More impatient individuals have poorer overall diet quality.

Supplementary Table S2. Summary of selected studies on dietary diversity estimates in Madagascar

Study	Location	Participants	Sample size	Dietary intake estimation		Dietary Diversity Indicator
				Period	Food groups	
[71]	Sahalava and Antsororokavo districts/ Urban	Children aged 6–23 months	702	24-hour	8	Non-breastfed infants (6–23 months)<br>Dietary Diversity Score (DDS): 4.4±1.2<br>MMDA[a]: 71±14
[72]	Moramanga and Morondava	Children aged 6–59 months	1824	24-hour	7	DDS: < 4 (42–48%)
[73]	Amoron'i Mania region	Non-pregnant mothers	670	24-hour	10	DDS: <5
[74]	Northeastern Madagascar/remote rainforest areas	Adults and children	719	24-hour	12	HDDS: 2.4–6.7
[75]	Eastern Madagascar/rural	Adults	150	24-hour dietary recall over 6-weeks	9	HDDS: 4
[34]	Andasibe, Alaotra-Mangoro Madagascar/rural	Caregivers	20	24-hour	9	DDS: 4.5±1.3
[37]	Southeastern coast/remote rainforest areas	Rice farmers	328	24-hour	13	IDDS: 3.22±0.99
[76]	Vakinankaratra /Central Highlands	Lowland rice-producing households	510	24-hour	12	HDRS[b]: 4.55±1.95<br>HDDS: 3.97±1.44

[a]The mean micronutrient density adequacy
[b] Household Dietary Richness Score
